# 

**Published:** 1887-11

**Authors:** 


### BOOKS RECEIVED.

---

OUTLINES FOR THE MANAGEMENT OF DIET. By EDWARD TUNNIS BRUEN, M. D.

WHAT TO DO IN CASES OF POISONING. By WILLIAM MURREL, M. D., F. R. C. P.

EIGHTEENTH ANNUAL REPORT OF THE STATE BOARD OF HEALTH OF MASSACHUSETTS.

A MANUAL OF TREATMENT BY MASSAGE AND METHODICAL MUSCLE EXERCISE. By JOSEPH SCHREIBER, M.D. Translated by WALTER MENDELSON, M.D.

TRANSACTIONS OF THE THIRTY-SEVENTH ANNUAL MEETING OF THE ILLINOIS STATE MEDICAL SOCIETY, CHICAGO, MAY, 1887.

SYPHILIS. By JONATHAN HUTCHINSON, F. R. S., LL. D.

SURGERY, ITS THEORY AND PRACTICE. By WILLIAM JOHNSON WALSHAM, F. R. C. S.

THE STUDENT'S GUIDE TO DISEASES OF THE EYE. By EDWARD NETTLESHIP, F. R. C. S.

DIFFERENTIAL DIAGNOSIS: A MANUAL OF COMPARATIVE SEMEIOLOGY OF THE MORE IMPORTANT DISEASES. By F. DE HAVILLAND HALL, M. D.

THE PRINCIPLES OF ANTISEPTIC METHODS APPLIED TO OBSTETRIC PRACTICE. By DR. PAUL BAR. Translated by HENRY D. FRY, M. D.

A HANDBOOK OF GENERAL AND OPERATIVE GYNÆCOLOGY. By DR. A. HEGAR and DR. R. KALTENBACH. Edited by E. H. GRANDIN, M. D. Vol. VI. of Cyclopedia of Obstetrics and Gynæcology.

DISEASES OF THE FEMALE URETHRA AND BLADDER. By F. WINCKLE, M. D.; and DISEASES OF THE VAGINA. By A. BREISKY, M. D. Edited by EGBERT H. GRANDIN, M. D. Vol. X. of Cyclopedia of Obstetrics and Gynæcology.

DRUITT'S SURGEON'S VADE-MECUM. A MANUAL OF MODERN SURGERY. Edited by STANLEY BOYD, M. B., B. S., LOND., F. R. C. S. ENG.

DIFFERENTIAL DIAGNOSIS OF THE DISEASES OF THE SKIN. For Students and Practitioners. By CONDICT W. CUTTER, M. S., M. D.

VASO-RENAL CHANGE *versus* BRIGHT'S DISEASE. By J. MILNER FOTHERGILL, M. D., EDIN.

INDEX CATALOGUE OF THE LIBRARY OF THE SURGEON-GENERAL'S OFFICE, U. S. ARMY, AUTHORS AND SUBJECTS. Vol. VIII. LEGREN-MEDICINE. (Naval.)

LESSONS IN GYNÆCOLOGY. By WILLIAM GODDELL, A. M., M. D.

A REFERENCE HANDBOOK OF THE MEDICAL SCIENCES. Edited by ALBERT H. BUCK, M. D.

A HANDBOOK OF GENERAL AND OPERATIVE GYNÆCOLOGY. By DR. A. HEGAR and DR. R. KALTENBACH. Vol. VII. of Cyclopædia of Obstetrics and Gynæcology.

DISEASES OF THE MAMMARY GLANDS. By TH. BILROTH, M. D. And NEW GROWTHS OF THE UTERUS. By A. GUSSEROW, M. D. Vol. IX. of Cyclopædia of Obstetrics and Gynæcology.

TREATISE ON HUMAN PHYSIOLOGY. For the Use of Students and Practitioners of Medicine. By HENRY C. CHAPMAN, M.D.

A MANUAL OF THE PHYSICAL DIAGNOSIS OF THORACIC DISEASES. By E. DARWIN HUDSON, JR., A.M., M.D.

OPERATIVE SURGERY IN THE CADAVER. By JASPER JEWETT GARMANY, A.M., M.D., F.R.C.S.

FUNCTIONAL NERVOUS DISEASES—THEIR CAUSES AND TREATMENT. By GEORGE T. STEVENS, M.D., Ph. G.

THE PRINCIPLES OF THEORETICAL CHEMISTRY, WITH SPECIAL REFERENCE TO THE CONSTITUTION OF CHEMICAL COMPOUNDS. By IRA REMSEN.

TRANSACTIONS OF THE ASSOCIATION OF AMERICAN PHYSICIANS. Second Session, June 2 and 3, 1887.

HEADACHES DUE TO EYE-STRAIN. By C. G. SAVAGE, M. D.

RENAL COLIC, PARASITIC AND CALCULOUS. By J. B. MARVIN, M. D.

HEREDITY AND TUBERCULOSIS. By FRANK DONALDSON, M. D.

A UNIQUE CASE OF BI-LATERAL ATHETOSIS. By C. H. HUGHES, M. D.

THE RELATION OF THE NERVOUS SYSTEM TO HÆMOPHILIA, MALARIAL HÆMATURIA, ETC. By C. H. HUGHES, M. D.

THE PREVENTION OF CHOLERA INFANTUM AND KINDRED DISEASES, AND POISONING BY CHEESE, MILK, ETC. By VICTOR C. VAUGHAN, M. D., Ph. D.

CONTRIBUTION A L'ETUDE CLINIQUE ET BACTERIOLOGIQUE DE LA FIEVRE JAUNE. Par le Dr. ANTONIO MATIENZO.

SURGICAL RELATIONS OF THE ILEOCÆCAL REGION. By J. McF. GASTON, M. D.

PATHOLOGY, DIAGNOSIS AND TREATMENT OF PERFORATION OF THE APPENDIX VERMIFORNIX. By J. McF. GASTON, M. D.

BIOLOGY OF TUMORS. By N. SENN, M. D., Ph. D.

REPORT OF THE ILLINOIS STATE BOARD OF HEALTH, QUARTERLY MEETING, JULY 8, 1887.

TRANSACTIONS OF THE PATHOLOGICAL SOCIETY OF PHILADELPHIA, Vol. XII., September, 1883, to July, 1885.

TRANSACTIONS OF THE MEDICAL AND CHIRURGICAL FACULTY OF THE STATE OF MARYLAND, APRIL, 1887.

TRANSACTIONS OF THE AMERICAN OTOLOGICAL SOCIETY, JULY 19, 1887.

TRANSACTIONS OF THE MEDICAL SOCIETY OF THE STATE OF WEST VIRGINIA, 1887.

TRANSACTIONS OF THE TEXAS STATE MEDICAL ASSOCIATION, 1887.

TRANSACTIONS OF THE MEDICAL ASSOCIATION OF THE STATE OF MISSOURI, 1887.

REPORT ON IMPROVED METHODS OF SEWAGE DISPOSAL AND WATER SUPPLIES. By C. W. CHANCELLOR, M. D.

Volume LV.] NOVEMBER, 1887. [Number 5.

LIBRARY
INDEXED
S.G.O.

### The CHICAGO MEDICAL JOURNAL AND EXAMINER

EDITOR:
S. J. JONES, M.D., LL.D

COLLABORATORS:

CHICAGO:

PROFESSOR E. ANDREWS, M.D., LL.D.

PROFESSOR J. BARTLETT, M.D.

PROFESSOR W. T. BELFIELD, M.D.

PROFESSOR F. BILLINGS, M.D.

PROFESSOR W. H. BYFORD, A.M., M.D.

PROFESSOR L. CURTIS, A.M., M.D.

PROFESSOR N. S. DAVIS, JR., A.M., M.D.

PROFESSOR O. C. DEWOLF, A.M., M.D.

PROFESSOR E. C. DUDLEY, A.B., M.D.

PROFESSOR C. FENGER, M.D.

PROFESSOR J. N. HYDE, A.M., M.D.

PROFESSOR E. F. INGALS, A.M., M.D.

PROFESSOR W. W. JAGGARD, A.M., M.D.

PROFESSOR F. S. JOHNSON, A.M., M.D.

PROFESSOR H. A. JOHNSON, M.D., LL.D.

PROFESSOR C. W. PURDY, M.D.

PROFESSOR C. G. SMITH, A.M., M.D.

PROFESSOR E. WING, A.M., M.D.

MILWAUKEE, WIS.

PROFESSOR N. SENN, M.D.

WASHINGTON, D. C.:
S. O. RICHEY, M.D.

UNITED STATES ARMY:
SURGEON D. L. HUNTINGTON, M.D.

MARINE HOSPITAL SERVICE:
SURGEON S. T. ARMSTRONG, M.D.

UNITED STATES NAVY:
MEDICAL DIRECTOR J. M. BROWNE, M.D.
MEDICAL DIRECTOR A. L. GIHON, A.M., M.D

